# Case Report: Cryptococcal eosinophilic meningitis in a patient with Hodgkin lymphoma

**DOI:** 10.3389/fneur.2022.898525

**Published:** 2022-09-07

**Authors:** Fang Zhang, Yuchen Li, Huijun Shen, Jie Tao, Jie Wang

**Affiliations:** ^1^Department of Neurology, First Hospital of Shanxi Medical University, Taiyuan, China; ^2^Graduate School, Shanxi Medical University, Taiyuan, China; ^3^Department of Hematology, First Hospital of Shanxi Medical University, Taiyuan, China

**Keywords:** cryptococcal meningitis, eosinophilic meningitis, eosinophilia, Hodgkin lymphoma, cryptococcal lymphadenitis

## Abstract

Cryptococcal meningitis is the most common fungal meningitis in clinical practice. It primarily occurs in immunocompromised people and is typically associated with human immunodeficiency virus (HIV) infection. In rare cases, it is associated with Hodgkin lymphoma (HL). Eosinophilic meningitis (EM) is characterized by increased eosinophils in the cerebrospinal fluid (CSF) and is often caused by a parasitic infection of the central nervous system (CNS). EM caused by cryptococcal infection is rare; only four cases have been reported in the past 30 years. Here, we report a case of cryptococcal meningitis in a patient with HL who presented with an atypical eosinophil-predominant CSF cytology response. The patient's eosinophil proportion reached 91%; a proportion this high has not been reported previously and may be associated with HL. After antifungal therapy and tumor chemotherapy, the proportion of eosinophils decreased significantly. This case shows that cryptococcal meningitis and HL may be simultaneously contributing to CSF eosinophilia. HL should be considered in patients with eosinophilic cryptococcal meningitis and multiple adenopathies.

## Introduction

Cryptococcal meningitis, caused by cryptococcal infection of the central nervous system (CNS), is the most common fungal meningitis in clinical practice. Most patients present with characteristic manifestations of meningitis, including headache, fever, and vomiting (1). The disease typically has a high mortality rate and a poor prognosis (2). Cryptococcal meningitis primarily occurs in immunocompromised patients and is typically associated with human immunodeficiency virus (HIV) infection. Hodgkin lymphoma (HL) is also considered a risk factor, but the association of both conditions is relatively rare (3, 4).

HL is a hematological malignancy, composed by large dysplastic mononuclear and multinucleated cells surrounded by a variable mixture of inflammatory cells. Peripheral adenopathies are usually the first manifestation of HL. The disease is categorized as classic HL (cHL) or nodular lymphocyte-predominant HL (NLPHL) (5). The former is subdivided into 4 histologic subtypes (Nodular sclerosis cHL, Mixed-cellularity cHL, Lymphocyte-rich cHL and Lymphocyte-depleted cHL). Over 90% of the cases are cHL, which behaves as an aggressive tumor; however, lymphocyte-rich cHL manifests with indolent biological nature in most instances and can be effectively treated with modern combination chemotherapy regimens (5). *Cryptococcus* infection in patients with HL occurs at ~2.7% over 10 years and can lead to increased mortality (6).

Eosinophilic meningitis (EM), a disease characterized by increased eosinophils in the cerebrospinal fluid (CSF), is often caused by a parasitic infection of the CNS (7). EM caused by cryptococcal meningitis is rare. To our knowledge, only four cases have been reported in the past 30 years (8–11). A proportion of eosinophils as high as 91% presented in this case has not been reported, and such a high proportion may be associated with HL, which was reported to have a correlation with CSF eosinophilia as well (12–14).

Here, we report a case of cryptococcal meningitis in a patient with HL who presented with an atypical eosinophil-predominant CSF cytology response. After antifungal therapy and tumor chemotherapy, the proportion of eosinophils decreased significantly.

## Case report

**Figure 1:**
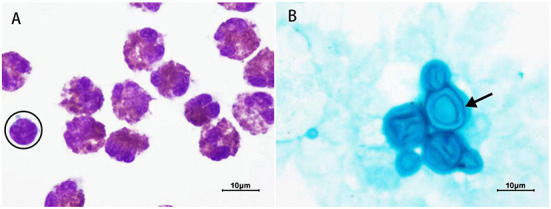
Results of cerebrospinal fluid (CSF) staining. **(A)** Wright-Giemsa staining (1,000 X). All cells are eosinophils except the one surrounded by a black circle, which is a lymphocyte. **(B)** Alcian blue staining (1,000 X). Black arrow = *Cryptococcus* with an intact capsule. The capsule of *Cryptococcus* is dark blue and the thallus is light blue.

A previously healthy 20-year-old male patient presented with intermittent headaches, nausea and vomiting for 13 days, which had worsened in the previous 2 days. He also presented fever the day before the hospital admission. He took no regular medication. Physical examination revealed cervical rigidity, lymph node enlargements on the right side of the neck. Vital signs (body temperature, pulse rate, respiration rate, and blood pressure) were normal. Relevant laboratory tests and examinations were performed after admission. Complete blood count was normal (Hb 130 g/L, Leucocytes 3.9 × 10^9^/L, with 1.4 × 10^9^ lymphocytes/L, 1.6 × 10^9^ neutrophils/L, 0.5 × 10^9^ eosinophils/L, and 0.4 × 10^9^ monocytes/L, and platelets 125 × 10^9^/L), and the patient tested negative for HIV. Brain magnetic resonance imaging (MRI) revealed hyperintensity in the bilateral frontopolar and parietal cortex and sulcus. A lumbar puncture revealed elevated CSF opening pressure (>330 mmH_2_O), decreased glucose (1.19 mmol/L), elevated protein (0.96 g/L), and pleocytosis of 358 × 10^6^ leucocytes/L (91% eosinophils, 4% lymphocytes, 4% monocytes, and 1% plasma cells) (Figure 1A). Alcian blue staining revealed *Cryptococcus* (Figure 1B), and the CSF culture grew *Cryptococcus neoformans* resistant to fluconazole. To search for the cause of eosinophilia, metagenomic next-generation sequencing (m-NGS) of the CSF was performed, and only *Cryptococcus neoformans* (sequence number: 1427) was identified; no parasites or other pathogens were found. The patient was diagnosed with cryptococcal meningitis on the basis of his clinical symptoms and signs combined with the presence of *Cryptococcus* in the CSF. The following examinations were performed to identify the cause of lymph node enlargements. Color Doppler ultrasound of the superficial lymph nodes revealed multiple lymph node enlargements in the bilateral neck, axilla, and right groin. Chest computed tomography (CT) showed multiple lymph node enlargements on both sides of the neck, mediastinum, and lung hilum. A biopsy of the cervical lymph node was performed, and the pathological diagnosis was lymphocyte-rich cHL. An 18F-FDG PET-CT was performed and documented hypermetabolic activity on supra- and infra-diaphragmatic adenopathies, spleen and bone, corresponding to the stage IV of the Lugano classification (15). CSF analysis by flow cytometry showed no malignant cells.

**Figure 2:**
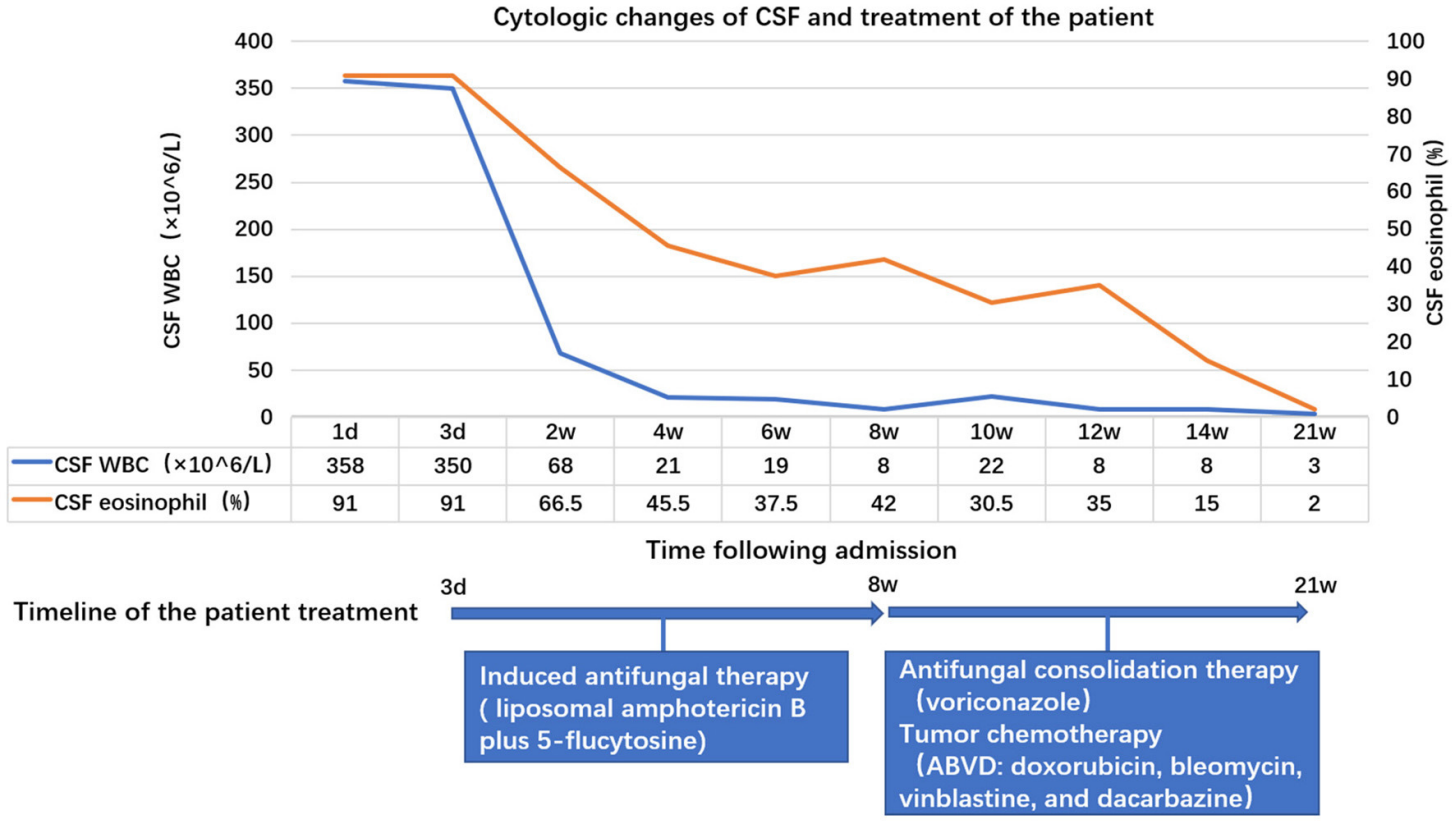
Changes in the leucocyte count and proportion of eosinophils in the cerebrospinal fluid (CSF) with treatment. On the first day of admission, the leukocyte count was 358 × 10^6^/L (91% eosinophils), which decreased to 8 × 10^6^/L (42% eosinophils) after about 8 weeks of antifungal induction therapy, and to 3 × 10^6^/L (2% eosinophils) after 13 weeks of antifungal consolidation therapy combined with tumor chemotherapy.

Treatment for HL may suppress immunity and thus aggravate cryptococcal infection, and lymphocyte-rich cHL has an indolent biological nature; therefore, we first administered antifungal induction therapy (liposomal amphotericin B plus 5-flucytosine) and performed successive lumbar punctures to control the high CSF opening pressure. After 8 weeks, the symptoms of meningitis significantly improved. The CSF leukocyte count (8 × 10^6^/L) and proportion of eosinophils (42%) were reduced, and a CSF culture was negative. The patient thus initiated antifungal consolidation therapy with voriconazole and was transferred to the hematology department to start chemotherapy with the ABVD protocol (doxorubicin, bleomycin, vinblastine, and dacarbazine). At the present moment, the patient has received antifungal consolidation therapy for 13 weeks and 4 cycles of ABVD and he is asymptomatic. CSF culture is still negative, and the leucocyte count and proportion of eosinophils in the CSF have decreased gradually with treatment (Figure 2).

## Discussion

Cryptococcal meningitis primarily occurs in immunocompromised hosts, most commonly HIV-infected patients. Other high-risk patient groups include patients receiving immunosuppressive therapies, patients with malignant tumors (including HL), solid organ transplant recipients, and patients with rheumatic diseases (3, 4). Our patient was HIV seronegative, with no history of immunosuppressive drugs, and had no other diseases. The immunosuppression associated to the HL contributed for the Cryptococcus infection. Studies have shown that ~16–19% of cryptococcal meningitis patients without HIV have malignant neoplasms (16). A large retrospective review of 583 patients with cryptococcal infection associated with malignancies from 1970 to 2014 found that 52 (9%) cases were associated with HL (4).

Previous studies have shown that risk factors for cryptococcal infection in patients with HL include a history of HL of ≥12 months, stage IV disease, absolute lymphopenia in the peripheral blood, and extensive pretreatment with chemotherapeutic agents (6, 17). In this case, the patient had no history of malignancies, no decrease in lymphocyte count, and no chemotherapeutic treatment before infection. The only identified risk factor for cryptococcal infection was stage IV disease. Previous studies have also shown that the host's defense against cryptococcal infection mainly relies on T cell immunity (18). Compared with healthy individuals, the number of T cells in HL patients is reduced, and the proliferation ability of T cells is significantly decreased (19), which partially explains why HL patients present a higher risk to be infected with *Cryptococcus*.

In addition, it is critical to identify HL and cryptococcal lymphadenitis in patients with cryptococcal meningitis accompanied by multiple lymph node enlargements. The most common sites of *Cryptococcus* infection are the CNS and the lungs (20). However, many reports (20–22) have demonstrated that *Cryptococcus* can infect the lymph nodes and lead to cryptococcal lymphadenitis, of which the clinical manifestations are very similar to lymphoma, including lymph node enlargements in the neck, supraclavicular region, mediastinum, and groin as well as splenomegaly. Biopsy is the gold standard to distinguish these two diseases (21, 22). To exclude the presence of a HL or other malignancy, it is recommended to routinely perform a lymph node biopsy in all patients with cryptococcal meningitis and adenopathies.

Eosinophilic meningitis (EM) is defined by the presence of 10 or more eosinophils/uL in the CSF or eosinophilia of at least 10% of the total CSF leukocyte count (23). The etiology can be divided into infectious and non-infectious causes (7, 23). Parasitic infection is the most common form of infectious EM. Other rare pathogens include fungi, Streptococcus, coxsackieviruses, and Rickettsia. Among pathogenic fungi, *Coccidioides* is the most common, whereas *Cryptococcus* is relatively rare. Non-infectious factors include tumors, drugs, toxins, and ventriculoperitoneal shunts.

EM caused by cryptococcal meningitis is rare. As mentioned above, only four cases have been reported in the past 30 years (Table 1). All patients had a significant medical history: one patient had been submitted to thymectomy and combined irradiation and chemotherapy; one patient had had angioimmunoblastic T-cell lymphoma; one patient had asthma, perennial rhinitis, atopic dermatitis and high-dose steroid therapy; the other patient had sarcoidosis (8–11). The initial proportion of eosinophils in CSF of three patients was 50, 12, and 76%, respectively; exact data is not available for the other patient. Eosinophilia totally resolved after antifungal therapy alone in three patients. In the other case, the eosinophils in the CSF decreased from 76 to 64% after 8 days of antifungal therapy, but no further CSF data is available. In the case we now report, the eosinophils in the CSF decreased from 91 to 42% after antifungal therapy alone. No parasites or other pathogens were found, and the patient had no history of taking special drugs or toxins. Therefore, *Cryptococcus* infection was considered a possible cause of EM. In addition, the proportion of eosinophils was as high as 91% in our case, suggesting that other factors may have been involved in eosinophilia.

**Table 1:** Summary of the clinical cases of cryptococcal meningitis with CSF eosinophilia published in the last 30 years.

**Refere nces**	**Gender**	**Age (years)**	**Past medical history**	**Manifestations**	**CSF leukocytes (× 10^6^/L)**	**CSF eosinophils (%)**	**Treatment**	**Outcome**
Schmidt et al. (8)	F	43	Thymectomy and multiple courses of combined irradiation and chemotherapy	Progressive waste, headache, fever, dysphagia and confusion	237	50	Fluconazole	Recovered well without neurological sequelae. CSF eosinophilia resolved after 11 months of therapy.
Grosse et al. (9)	F	64	Angioimmunoblastic T-cell lymphoma	Headache, fatigue and inattentiveness	582	12	Amphotericin, flucytosine and fluconazole	Recovered well without neurological sequelae. CSF eosinophilia resolved after 7 weeks of therapy.
Pfeffer et al. (10)	F	22	Asthma, perennial rhinitis, atopic dermatitis and high-dose steroid therapy	Headache, fever and encephalopathy	33	n.a.	Amphotericin, flucytosine and fluconazole	Recovered well without neurological sequelae. CSF eosinophilia resolved after 3 months of therapy.
Hadid et al. (11)	M	51	sarcoidosis	Headache, neck stiffness and photophobia	1,826	76	Amphotericin, flucytosine and fluconazole	Recovered well without neurological sequelae. CSF eosinophils reduced to 64% after 8 days of therapy.

F, female; M, male; n.a., Not available; CSF, Cerebrospinal fluid.

Current studies have shown that HL can cause peripheral blood eosinophilia with an incidence of ~15% (24). However, few studies have investigated the association between HL and CSF eosinophilia, except in the rare cases of HL meningeal invasion, which can present with EM (12–14). The diagnosis involvement of the meninges by HL relies on meningeal biopsy and detection of malignant cells in the CSF (13). In this case, the patient did not undergo any meningeal biopsy. Taking into account that the several CSF cytological examinations and the flow cytometry analysis did not reveal any malignant cells and that the symptoms of meningitis significantly improved without intrathecal chemotherapy, a meningeal involvement by HL was considered unlikely. However, the contribution of HL to the CSF eosinophilia cannot be excluded as the proportion of eosinophils was still high (42%) after 8 weeks of antifungal therapy alone, despite the negative CSF culture, and it decreased to 2% after the chemotherapy initiation. The specific mechanism requires further studies.

In conclusion, cryptococcal meningitis and HL may be simultaneously contributing to CSF eosinophilia. HL should be considered in patients with eosinophilic cryptococcal meningitis and multiple adenopathies. Early diagnosis and treatment of eosinophilic cryptococcal meningitis and the underlying immunodeficiency disease is essential for improving the clinical outcomes of these patients.

## Data availability statement

The original contributions presented in the study are included in the article/supplementary material, further inquiries can be directed to the corresponding author.

## Ethics statement

Written informed consent was obtained from the individual(s) for the publication of any potentially identifiable images or data included in this article.

## Author contributions

FZ, YL, HS, JT, and JW examined and treated the patient. FZ contributed to the conception and writing of the first draft of the manuscript. YL and HS collected the data. JW contributed to the critical revision of the manuscript. All authors contributed to the article and approved the submitted version.

## Conflict of interest

The authors declare that the research was conducted in the absence of any commercial or financial relationships that could be construed as a potential conflict of interest.

## Publisher's note

All claims expressed in this article are solely those of the authors and do not necessarily represent those of their affiliated organizations, or those of the publisher, the editors and the reviewers. Any product that may be evaluated in this article, or claim that may be made by its manufacturer, is not guaranteed or endorsed by the publisher.

## References

[1] Nathan CL Emmert BE Nelson E Berger JR. CNS fungal infections: a review. J Neurol Sci. (2021) 422:117325. 10.1016/j.jns.2021.117325 33516057

[2] Pasquier E Kunda J De Beaudrap P Loyse A Temfack E Molloy SF. Long-term mortality and disability in cryptococcal meningitis: a systematic literature review. Clin Infect Dis. (2018) 66:1122–32. 10.1093/cid/cix870 29028957

[3] Williamson PR Jarvis JN Panackal AA Fisher MC Molloy SF Loyse A. Cryptococcal meningitis: epidemiology, immunology, diagnosis and therapy. Nat Rev Neurol. (2017) 13:13–24. 10.1038/nrneurol.2016.167 27886201

[4] Schmalzle SA Buchwald UK Gilliam BL Riedel DJ. *Cryptococcus neoformans* infection in malignancy. Mycoses. (2016) 59:542–52. 10.1111/myc.12496 26932366

[5] Shanbhag S Ambinder RF. Hodgkin lymphoma: a review and update on recent progress. CA Cancer J Clin. (2018) 68:116–32. 10.3322/caac.21438 29194581 PMC5842098

[6] Korfel A Menssen HD Schwartz S Thiel E. Cryptococcosis in Hodgkin's disease: description of two cases and review of the literature. Ann Hematol. (1998) 76:283–6. 10.1007/s002770050403 9692818

[7] Graeff-Teixeira C da Silva AC Yoshimura K. Update on eosinophilic meningoencephalitis and its clinical relevance. Clin Microbiol Rev. (2009) 22:322–48. 10.1128/CMR.00044-08 19366917 PMC2668237

[8] Schmidt S Reiter-Owona I Hotz M Mewes J Biniek R. An unusual case of central nervous system cryptococcosis. Clin Neurol Neurosurg. (1995) 97:23–7. 10.1016/0303-8467(94)00063-c 7788968

[9] Grosse P Schulz J Schmierer K. Diagnostic pitfalls in eosinophilic cryptococcal meningoencephalitis. Lancet Neurol. (2003) 2:512. 10.1016/s1474-4422(03)00488-5 12878440

[10] Pfeffer PE Sen A Das S Sheaff M Sivaramakrishnan A Simcock DE. Eosinophilia, meningitis and pulmonary nodules in a young woman. Thorax. (2010) 65:1066–85. 10.1136/thx.2010.140350 20889522

[11] Hadid H Nona P Usman M Paje D. Cryptococcal eosinophilic meningitis in a patient with sarcoidosis. BMJ Case Rep. (2015) 2015:bcr2015212765. 10.1136/bcr-2015-212765 26682840 PMC4691860

[12] Strayer DR Bender RA. Eosinophilic meningitis complicating Hodgkin's disease. A report of a case and review of the literature. Cancer. (1977) 40:406–9. 10.1002/1097-0142(197707)40:1<406::aid-cncr2820400158>3.0.co;2-n 69484

[13] Hauke RJ Tarantolo SR Bashir RM Moravec D Bierman PJ. Central nervous system Hodgkin's disease relapsing with eosinophilic pleocytosis. Leuk Lymphoma. (1996) 21:173–5. 10.3109/10428199609067596 8907286

[14] Sachdeva MU Suri V Malhotra P Srinivasan R. Cerebrospinal fluid infiltration in Hodgkin lymphoma: a case report. Acta Cytol. (2008) 52:623–6. 10.1159/000325609 18833829

[15] Cheson BD Fisher RI Barrington SF Cavalli F Schwartz LH Zucca E. Recommendations for initial evaluation, staging, and response assessment of Hodgkin and non-Hodgkin lymphoma: the Lugano classification. J Clin Oncol. (2014) 32:3059–68. 10.1200/JCO.2013.54.8800 25113753 PMC4979083

[16] Beardsley J Sorrell TC Chen SC. Central nervous system cryptococcal infections in non-HIV infected patients. J Fungi. (2019) 5:71. 10.3390/jof5030071 31382367 PMC6787755

[17] Dioverti MV Parikh SA Osmon DR Habermann TM Tande AJ. *Cryptococcus neoformans* infections in patients with lymphoproliferative neoplasms. Leuk Lymphoma. (2019) 60:920–26. 10.1080/10428194.2018.1508666 30188226

[18] Segal BH Bow EJ Menichetti F. Fungal infections in nontransplant patients with hematologic malignancies. Infect Dis Clin North Am. (2002) 16:935–vii. 10.1016/s0891-5520(02)00043-0 12512188

[19] Fisher RI DeVita VT Jr Bostick F Vanhaelen C Howser DM Hubbard SM. Persistent immunologic abnormalities in long-term survivors of advanced Hodgkin's disease. Ann Intern Med. (1980) 92:595–9. 10.7326/0003-4819-92-5-595 6992672

[20] Coskun ZU Mathews D Weatherall P Skiest D Oz OK. Cryptococcal lymphadenitis and massive splenomegaly in an immunocompromised patient. Clin Nucl Med. (2007) 32:314–6. 10.1097/01.rlu.0000257201.95620.ba 17413585

[21] Srinivasan R Gupta N Shifa R Malhotra P Rajwanshi A Chakrabarti A. Cryptococcal lymphadenitis diagnosed by fine needle aspiration cytology: a review of 15 cases. Acta Cytol. (2010) 54:1–4. 10.1159/000324958 20306981

[22] Chen J Zhang MJ Ge XH Liu YH Jiang T Li J. Disseminated cryptococcosis with multiple and mediastinal lymph node enlargement and lung involvement in an immunocompetent child. Int J Physiol Pathophysiol Pharmacol. (2019) 11:293–6. 31993105 PMC6971505

[23] Lo Re V 3rd Gluckman SJ. Eosinophilic meningitis. Am J Med. (2003) 114:217–23. 10.1016/s0002-9343(02)01495-x 12637136

[24] Ramirez GA Yacoub MR Ripa M Mannina D Cariddi A Saporiti N. Eosinophils from physiology to disease: a comprehensive review. Biomed Res Int. (2018) 2018:9095275. 10.1155/2018/9095275 29619379 PMC5829361

